# Comparative genomic analysis of *Citrobacter* sp. XT1-2-2 reveals insights into the molecular mechanism of microbial immobilization of heavy metals

**DOI:** 10.1186/s12864-022-09069-4

**Published:** 2022-12-19

**Authors:** Shiping Shan, Wei Cheng, Yilu Li, Min Zhang, Zhudong Liu, Yushuang Wang, Xiaowu Wei, Zujiao Fu, Shandong Wu, Dongxia Du, Zhaohui Guo

**Affiliations:** 1 Hunan Institute of Microbiology, 410009 Changsha, Hunan China; 2 Hunan Engineering and Technology Research Center of Agricultural Microbiology Application, 410009 Changsha, Hunan China

**Keywords:** *Citrobacter*, Cadmium, Microbial immobilization, Rice, Sulfate reduction pathway

## Abstract

**Background:**

In our previous study, *Citrobacter* sp. XT1-2-2 was isolated from high cadmium-contaminated soils, and demonstrated an excellent ability to decrease the bioavailability of cadmium in the soil and inhibit cadmium uptake in rice. In addition, the strain XT1-2-2 could significantly promote rice growth and increase rice biomass. Therefore, the strain XT1-2-2 shows great potential for remediation of cadmium -contaminated soils. However, the genome sequence of this organism has not been reported so far.

**Results:**

Here the basic characteristics and genetic diversity of the strain XT1-2-2 were described, together with the draft genome and comparative genomic results. The strain XT1-2-2 is 5040459 bp long with an average G + C content of 52.09%, and contains a total of 4801 genes. Putative genomic islands were predicted in the genome of *Citrobacter* sp. XT1-2-2. All genes of a complete set of sulfate reduction pathway and various putative heavy metal resistance genes in the genome were identified and analyzed.

**Conclusions:**

These analytical results provide insights into the genomic basis of microbial immobilization of heavy metals.

**Supplementary Information:**

The online version contains supplementary material available at 10.1186/s12864-022-09069-4.

## Background

The *Citrobacter* species belong to the domain *Bacteria* [1], the phylum *Proteobacteria* [2], the class *Gammaproteobacteria* [3], the order *Enterobacteria* [4], the *Enterobacteriaceae* family [5] and *Citrobacter* genus [6], and was introduced in 1932 by Werkman and Gillen [7]. The *Citrobacter* genus typically utilizes citric acid as the primary carbon source [8, 9]. *Citrobacter* species are commonly found in soil, water, sewage and food, sometimes exist as a normal flora in the gastrointestinal tract, also in human and animal feces, and sometimes as opportunistic pathogens isolated from clinical samples [10, 11].

*Citrobacter* sp. XT1-2-2 was isolated from high Cd-contaminated paddy soil. In our previous study, we found that the strain XT1-2-2 could tolerate a variety of heavy metals, and showed remarkable removal efficiency of Cd^2+^ in the solution compared with controls. Meanwhile, the strain could decrease the bioavailability of Cd in the soil and inhibit Cd uptake in rice plants. In addition, the strain could significantly promote rice growth and increase rice biomass [12]. These effects are mainly due to the strain's ability to reduce sulfate (SO_4_^2−^) to sulfide ions (S^2−^), and then sulfide ions (S^2−^) can combine with cadmium ions (Cd^2+^) existing in the soil to produce cadmium sulfide (CdS) precipitation, thereby converting the highly active cadmium ions (Cd^2+^) into residual cadmium sulfide (CdS), and then reduces the absorption and transport of cadmium by rice [13, 14]. Therefore, these characteristics made the strain XT1-2-2 strong potential for application to remediate Cd-contaminated paddy soils. However, the genome sequence and basic properties of this organism have not been reported so far. Here we report the high quality draft genomic information of the strain XT1-2-2 and conduct comparative genomic analysis with the other relevant reference sequenced genomes.

## Results

### Organism classification and characteristics

**Fig. 1:**
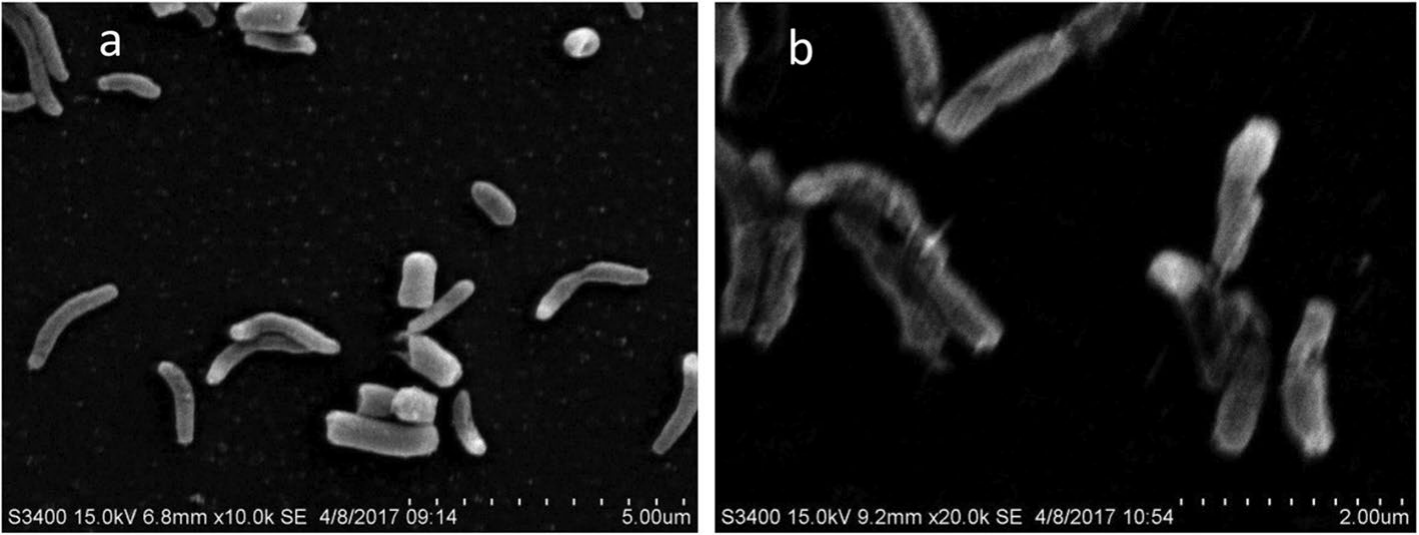
Scanning electron micrograph depicting effect of Cd on cellular morphology of *Citrobacter* sp. XT1-2-2. **a** *Citrobacter* sp. XT1-2-2 in absence of Cd^2+^. **b** *Citrobacter* sp. XT1-2-2 in presence of 100mg/L Cd^2+^

The strain XT1-2-2 is Gram-negative, facultatively anaerobic, non-sporulating, motile and rod-shaped (Fig. 1). The colonies are circular, smooth and opaque with a regular slick edge on SRB agar plates [13]. The strain XT1-2-2 is a non-pathogenic and free-living bacterium. Growth occurs at 15–40℃ and at pH 5–10. Optimal growth occurs at 30℃ and at pH 6–8. The basic characteristics and classification of the strain XT1-2-2 are shown in Table S1. The results of previous studies showed that the strain XT1-2-2 exhibited high resistance to a variety of heavy metals, and the MIC of the strain XT1-2-2 for Cd^2+^ was as high as 400 mg/L [12].

### SEM analysis

The scanning electron micrograph (SEM) analysis (Fig. 1) showed that cell shape was significantly influenced under high concentrations of Cd^2+^ of up to 100 mg/L. Compared with the control group (a), some cells in the treatment group (b) were twisted, lysed, or even broken. Oxidative damage and membrane permeability changes, caused by high Cd concentration, might be responsible for the cell morphology changes.

### TYGS analysis and phylogenetic relation

**Fig. 2:**
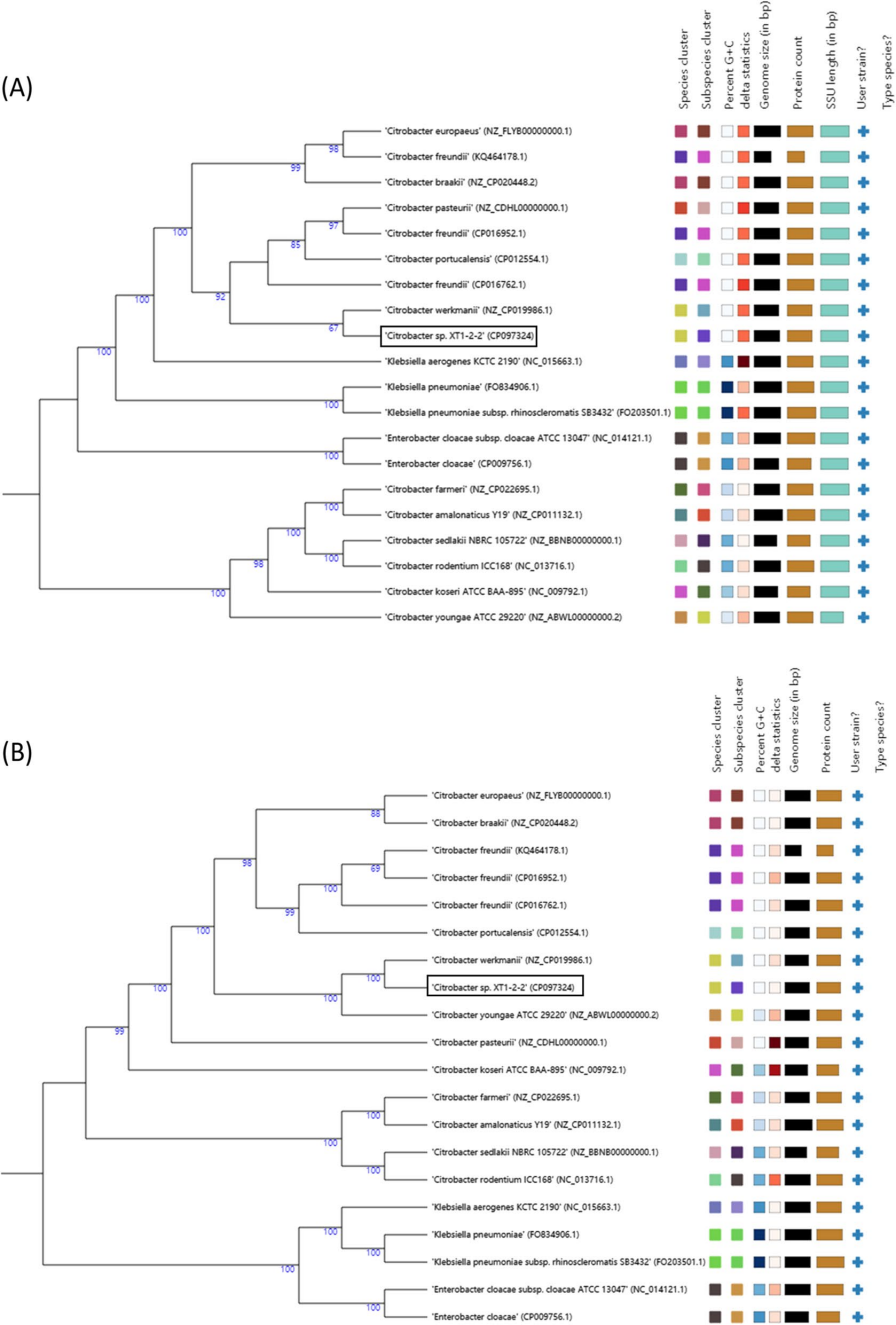
Genome BLAST Distance Phylogeny method (GBDP) for phylogenetic placement analysis using FastME 2.1.6.1 with 100 bootstrap values. **A** 16S rDNA gene sequence-based phylogeny of *Citrobacter* sp. XT1-2-2 with the closely related type strains and whole genomes with 93.5% average branch support. **B** Whole-genome sequence based phylogeny among the closely related type strains and whole genomes with 97.2% branch support. The numbers above branches represent the GBDP pseudo-bootstrap value, which is greater than 60%

The phylogenetic tree inferred from the intergenomic distance calculated from Genome BLAST Distance Phylogeny (GBDP) in the Type Strain Genome Server (TYGS) is shown in Fig. 2. Based on the 16S rDNA comparison, *Citrobacter* sp. XT1-2-2 is the closest relative to *Citrobacter werkmanii* BF-6 (CP019986.1) (Fig. 2). Similarly, the whole genome-based phylogeny also showed a cluster of the same species as the closest relatives of *Citrobacter* sp. XT1-2-2 (Fig. 2). All the *Citrobacter* species clustered together in a paraphyletic clade from the other type strains.

### Genome sequencing, annotation and features

**Fig. 3:**
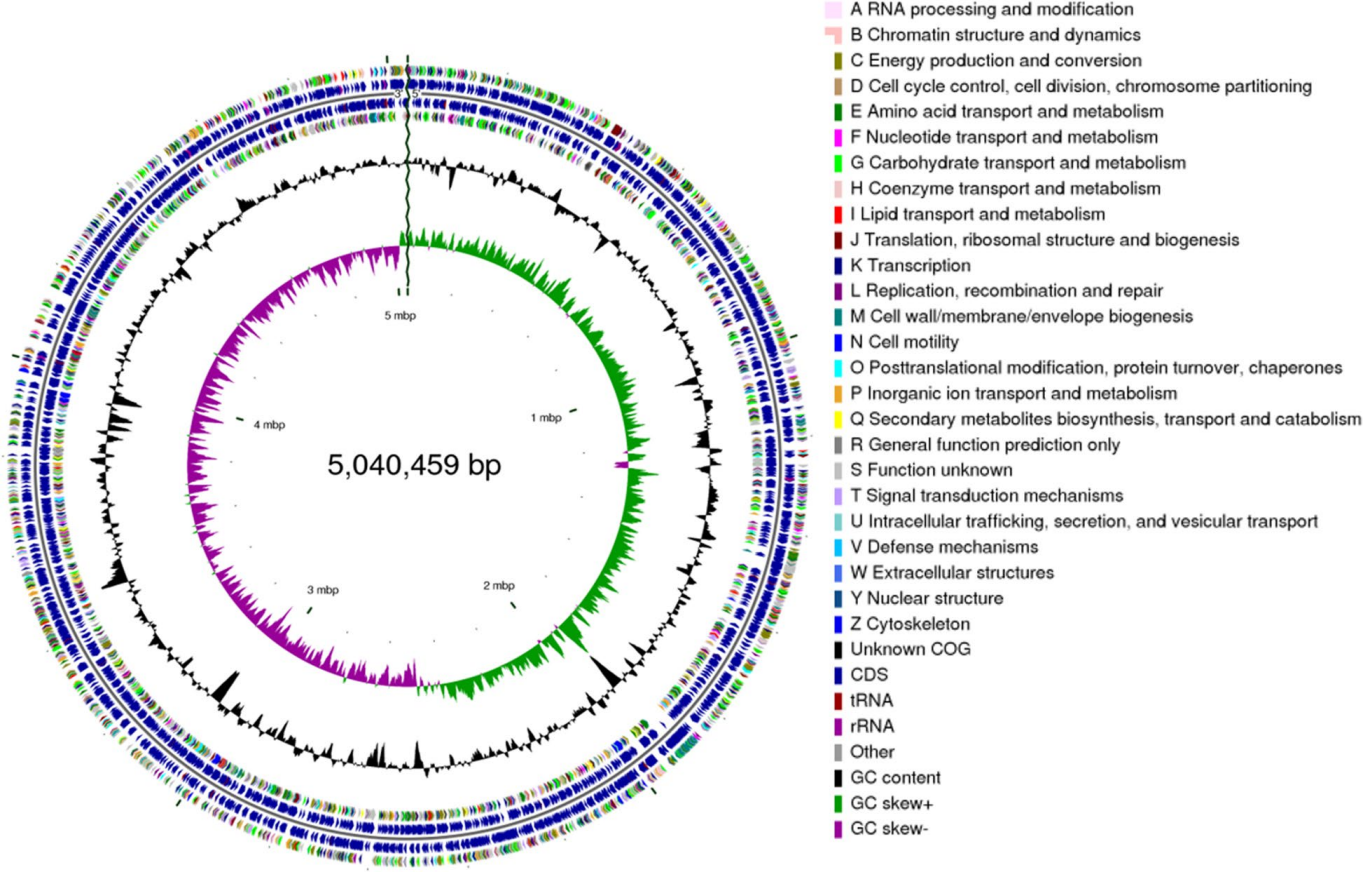
A graphical circular map of *Citrobacter* sp. XT1-2-2. From outside to center, rings 1, 4 show protein-coding genes colored by COG categories on forward/reverse strand; rings 2, 3 denote genes on forward/reverse strand; ring 5 shows G + C % content plot; ring 6 shows GC skew; the innermost ring shows the marker of genome size

**Fig. 4:**
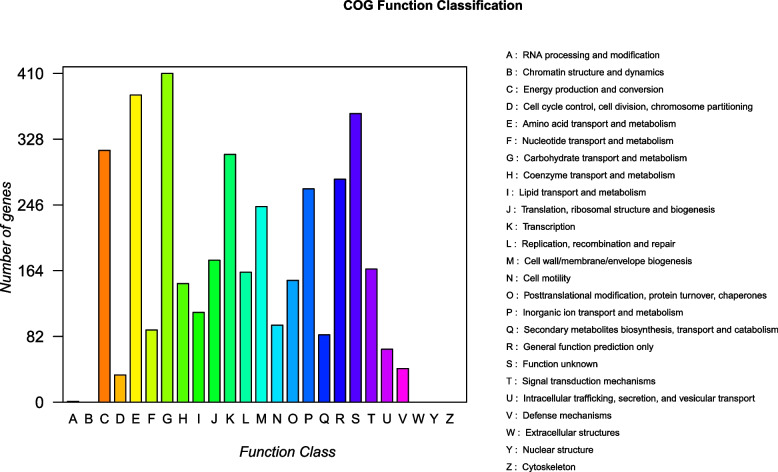
Number of genes associated with the 20 general COG functional categories. The gene number of the category B, W, Y, Z and X is zero

The strain XT1-2-2 was selected for sequencing particularly due to its multiple heavy metals resistance and heavy metal removal ability. Genome sequencing was performed by Shanghai Majorbio Bio-pharm Technology Co., Ltd (Shanghai, China). The project information is summarized in Table S2. The constructed standard shotgun library generated 165575 reads totaling 1164774594 bp and an average length of 7034.7 bp. The total size of the genome is 5,040,459 bp with 52.09% G + C content (Fig. 3). The genome properties and statistics are shown in Table S3. A total of 4801 genes, 4601 CDSs with protein, and 120 predicted RNA genes, including 84 tRNA, 25 rRNA and 11 ncRNA were predicted. In addition, 4383 (91.0%) genes are distributed into COG functional categories (Fig. 4).

### Identification of sulfate reduction pathway

**Fig. 5:**
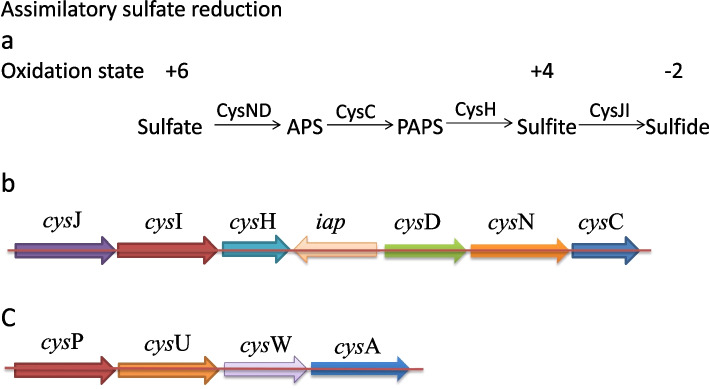
Putative sulfur metabolism pathway and assimilatory sulfate reduction genes in *Citrobacter* sp. XT1-2-2. **a** Putative sulfur metabolism pathway. **b** Assimilatory sulfate reduction gens in *Citrobacter* sp. XT1-2-2. c Putative sulfate transporter CysPUWA

According to the KEGG prediction analysis, the strain XT1-2-2 contains all genes of the complete set of sulfate reduction pathway (Fig. 5), including *cys*A, *cys*C, *cys*D, *cys*H, *cys*I, *cys*J, *cys*N, *cys*P, *cys*U, *cys*W. which provides the genomic basis for the strain to reduce sulfate (SO_4_^2−^) to sulfide (S^2−^) to form CdS precipitation, thereby reducing the uptake and transport of Cd^2+^ by rice. The basic information of sulfate reduction pathway genes including gene ID on chromosome, gene name, gene description has been analyzed, and heavy metal resistance genes have been already compared with the reference proteins in the swissprot database, and all the information has been shown in Table S4.

### Identification of heavy metal resistance genes

**Fig. 6:**
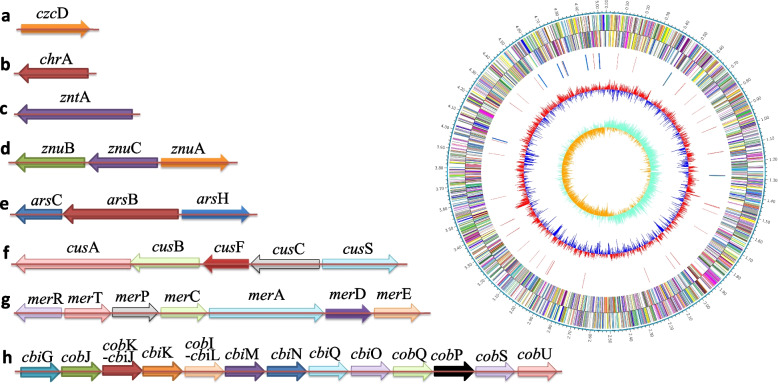
Heavy metal resistance genes distributed in *Citrobacter* sp. XT1-2-2. **a** Zinc or cadmium transporter, **b** Chromate transporter, **c** Zinc/ cadmium /mercury /lead-transporting ATPase, **d** Zinc ABC transporter permease, **e** Arsenical resistance protein, **f** copper resistance system, g Mercury transport system, h Cobalt ECF transporter complex

The results of previous studies showed that the strain XT1-2-2 could tolerate a variety of heavy metals (Cd^2+^, Pb^2+^, Zn^2+^, Mn^2+^ and Cr^6+^) and the removal rate of Cd^2+^ in solution is as high as 82.3 ± 2.1% within 240 min [12]. These results suggest that the strain XT1-2-2 has developed many evolutionary strategies to adapt the complex heavy metal pollution environment. According to the results of genome annotation, the strain XT1-2-2 contains multiple putative functional proteins, which are related to heavy metal resistance, including transporters, resistance proteins and metal reductases, and so on (Fig. 6). The basic information of heavy metal resistance genes including gene ID on chromosome, gene name, gene description has been analyzed, and heavy metal resistance genes have been already compared with the reference proteins in the swissprot database, and all the information has been shown in Table S5.

### Identification of the heavy metal resisitance genomic island

**Fig. 7:**
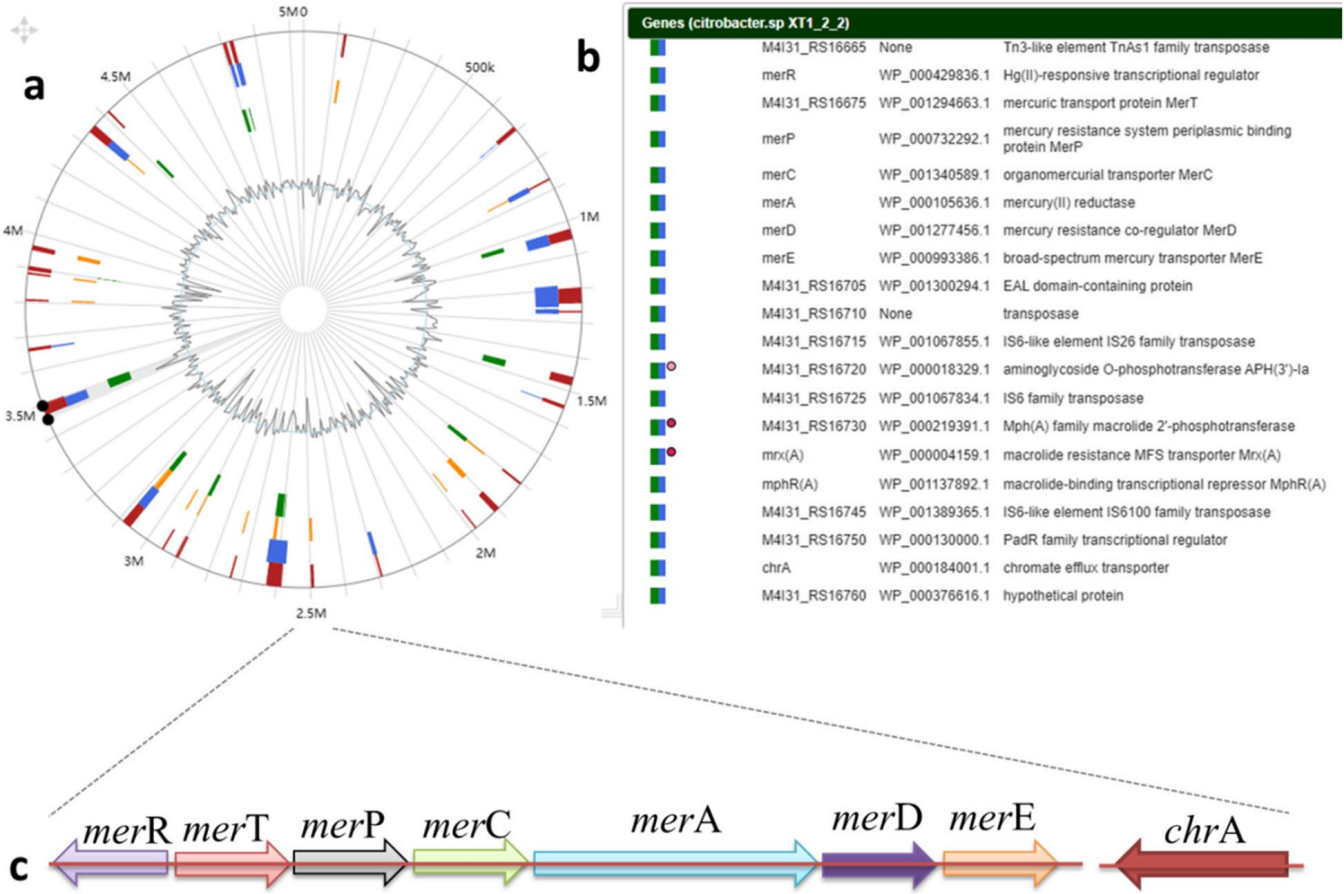
Identification of heavy metal resistance genomic island in the genome of *Citrobacter* sp. XT1-2-2. **a** The genomic islands were predicted by Island Viewer 4. Putative genomic islands predicted by the SIGI-HMM method (blue squares) or IslandPath-DIMOB method (yellow squares) or IslandPich (green squares). The integrated results are indicated by red squares. The inner ring indicated the G + C contents. **b** A vertical view of the genes and their description. **c** The gene arrangement of heavy metal resistance genes

The genomic islands in *Citrobacter* sp. XT1-2-2 were predicted by genome annotation combined with Island Viewer 4 software (Fig. 7), and all the genes (Table S6) on the genomic islands were further analyzed. Among all heavy metal resistance genes present on the genome, the membrane transporter *chr*A and mercury transport system (*mer*R, *mer*T, *mer*P, *mer*C, *mer*A, *mer*D, and *mer*E) were present on the same gene island. The region of the gene island ranging from nucleotide positions 3469026 to 3490287 were annotated as heavy metal resistance genomic island by SIGIHMM and IslandPath-DIMOB analysis.

### Features of the core and pan-genomes

**Fig. 8:**
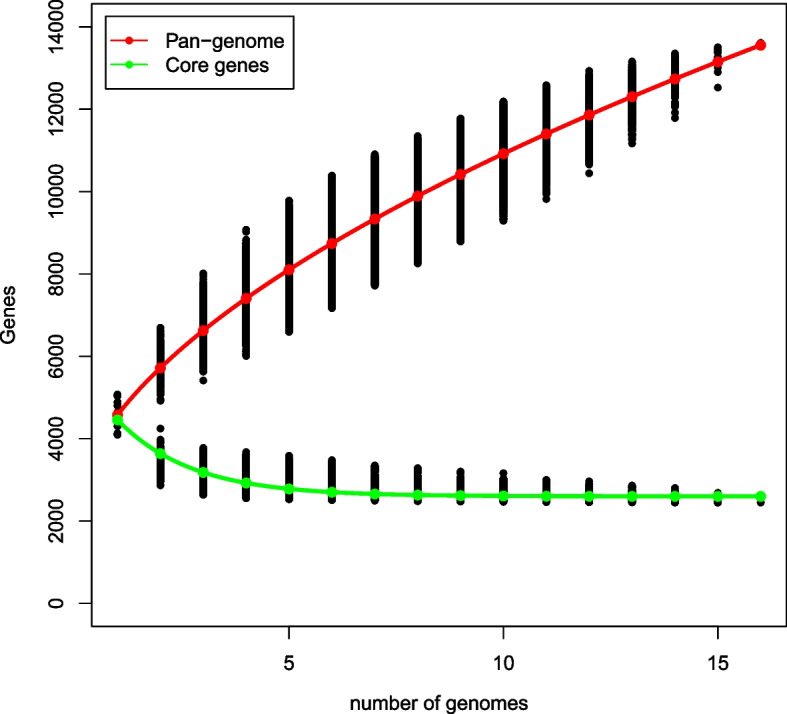
The *Citrobacter* core and pan-genome plotted were constructed for 16 genome sequences of *Citrobacter* related species

In order to assess genetic diversity, we constructed *Citrobacter* genus core and pan genomes and compared the gene content of *Citrobacter* sp. XT 1-2-2 with other relevant reference strains (Fig. 8). The basic information of the strains used for pan genome analysis has been indicated in Table S7, including the strain name, G + C content, number of proteins, genome size and the accession numbers of the *Citrobacter* species. From the alignment results, 13,614 gene families were found in 16 genomes, of which 2,449 genes constitute the core genome. The functional categories of the core gene families were further determined via the Cluster of Orthologous Group (COG) assignments among all the related species. The results showed that the core gene family presented an uneven distribution among functional categories (Fig. 4). We further analyzed the core, accessory and specific genes (Tables S8, S9 and S10), carefully checked the classification of the heavy metal resistance in the gene category, and the results are shown in Table S11.

### Comparative genomics analysis

**Fig. 9:**
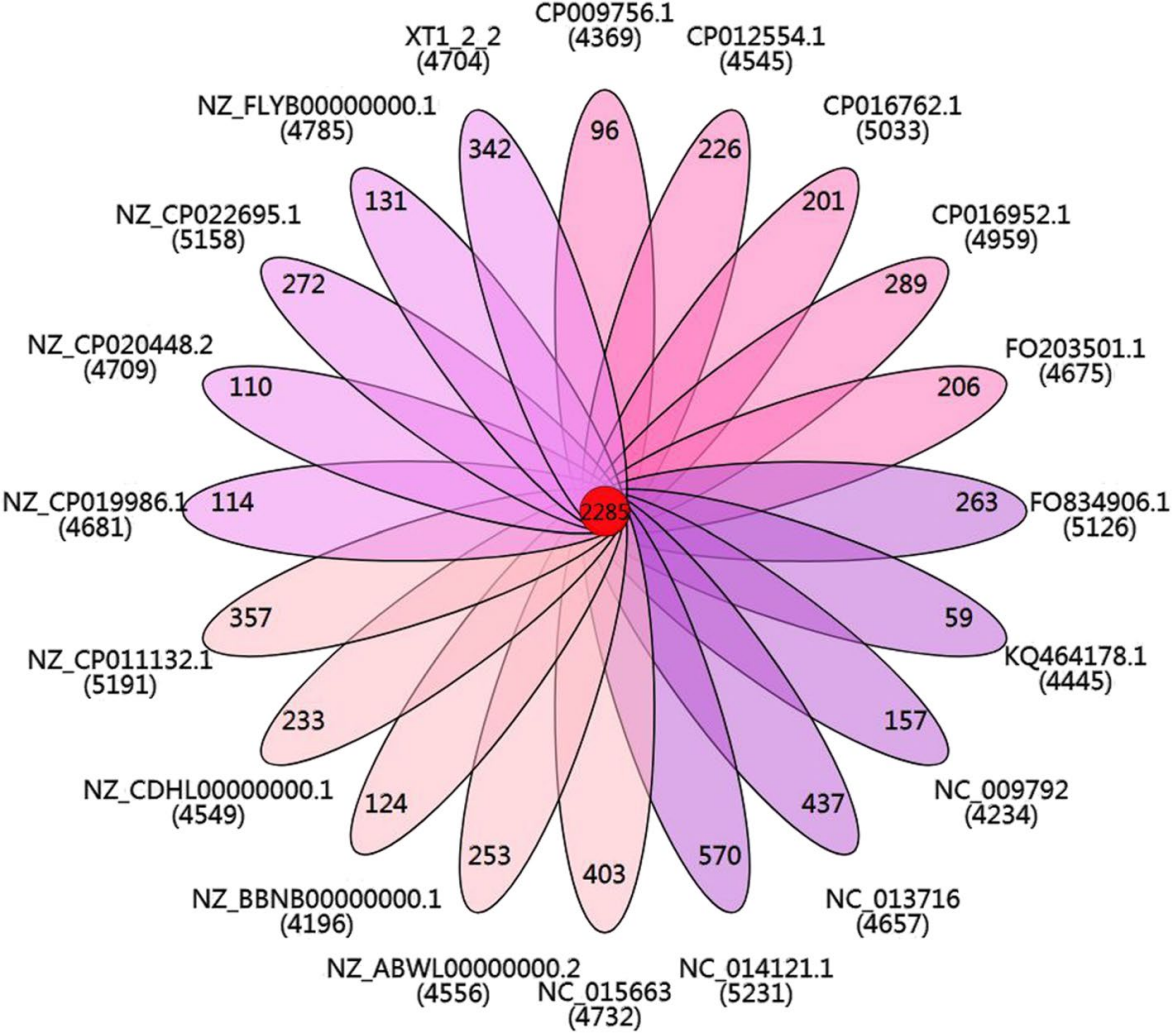
The Venn diagram depicting the core and unique genes between *Citrobacter* sp. XT1-2-2 and other 19 relevant reference species

The amino acid sequences of the involved twenty species were aligned via the OrthoMCL, and a certain threshold (E-Value: 1e-5, Percent Identity Cutoff: 0, Markov Inflation Index: 1.5) was selected for similarity clustering to obtain homologous genes. With the help of Venn diagram, the common and unique homologous genes between species are displayed intuitively. The strain XT1-2-2 shares 2285 proteins with the other genomes and has 342 specific proteins. The 2285 core genes include the genes in the whole sulfate reduction pathway and some of the heavy metal resistance genes (Fig. 9).

## **Discussion**

In this study, the complete genome of *Citrobacter* sp. XT1-2-2 was sequenced and comparative genomics analysis was also conducted with the other relevant reference sequenced genomes. In our previous study, the strain XT1-2-2 was isolated from high Cd-contaminated soils, and demonstrated an excellent ability to decrease the bioavailability of Cd in the soil and inhibit Cd uptake in rice. In addition, the strain XT1-2-2 could significantly promote rice growth and increase rice biomass. However, the genome sequence of this organism has not been reported so far.

The antigenic system of the Bethesna-Ballerup group bacteria was established by West and Edwards in 1954 [15]. This group of bacteria is now called *Citrobacter freundii* [16]. So far, *Citrobacter* genus contains eleven species: *Citrobacter freundii*, *Citrobacter koseri*, *Citrobacter amalonaticus*, *Citrobacter farmeri*, *Citrobacter youngae*, *Citrobacter braakii*, *Citrobacter werkmanii*, *Citrobacter sedlakii*, *Citrobacter rodentium*, *Citrobacter* genomospecies 10, *Citrobacter* genomospecies 11 [17, 18]. According to the results of TYGS analysis and phylogenetic relation, *Citrobacter* sp. XT1-2-2 is the closest relative to *Citrobacter werkmanii* BF-6 (CP019986.1) (Fig. 2). According to the physicochemical properties of these strains, some *Citrobacter* species immobilized biofilms were used to bioremediate heavy metal contaminated soils through an acid-type phosphatase enzymatic activity or their ability to accumulate heavy metals [19–21]. In this study, genome analysis of the strain XT1-2-2 revealed all genes of a complete set of sulfate reduction pathway according to the KEGG analysis (Fig. 5). The occurrence of metabolic pathways involves the following steps: (1) Sulfate (SO_4_^2−^) from outside is taken up into cells by putative sulfate transporter CysPUWA; (2) Sulfate (SO_4_^2−^) entering the cell is first acetylated to adenylylsulphate (APS) by sulfate adenylyltransferases CysN and CysD; (3) The resulting APS is then phosphorylated to phosphoadenylyl-sulphate (PAPS) by the APS kinase CysC; (4) The resulting PAPS is further reduced to sulfite (SO_3_^2−^) by PAPS reductase CysH; (5) The resulting sulfite (SO_3_^2−^) is finally reduced to sulfide (S^2−^) by sulfite reductase CysIJ [14]. The reason why the strain XT1-2-2 has a significant effect of removing cadmium is mainly because the strain generates sulfide (S^2−^) via the sulfur metabolism pathway, which can combine with Cd^2+^ in the soil to form the precipitated CdS, thereby reducing the uptake and transport of cadmium in the soil by rice plant.

Meanwhile, the strain XT1-2-2 also revealed various genes responsible for multiple heavy metal resistance (Fig. 6), which provided the genomic basis for the strain to adapt to the external complex harmful environment. CzcD is involved in resistance to the heavy metals Cd^2+^, Zn^2+^ and Co^2+^ [22]. The membrane transporter ChrA is responsible for the efflux of intracellular Cr(VI) from the cell [23]. Heavy metal-transporting ATPase (ZntA) is responsible for the efflux of Pb^2+^, Zn^2+^ and Cd^2+^ [24]. The metal ABC transport system (ZnuABC) are involved in Zn^2+^ uptake [25]. ArsB, ArsC, and ArsH proteins are involved in the functions of arsenical pump membrane protein, arsenate reductase and arsenical resistance protein, respectively [26]. Cus copper resistance system consists of CusCBA efflux pump, CusF periplasmic protein and CusS regulatory protein [27]. Mercury transport system (*mer* operon) encodes a group of proteins consisting of MerR mercury regulatory proteins, MerT, MerC, MerP mercury transport proteins and MerA, MerD, MerE mercury resistance proteins [28]. The Co^2+^ ECF transporter complex is involved in Co^2+^ resistance and transmembrane transport [29].

**Fig. 10:**
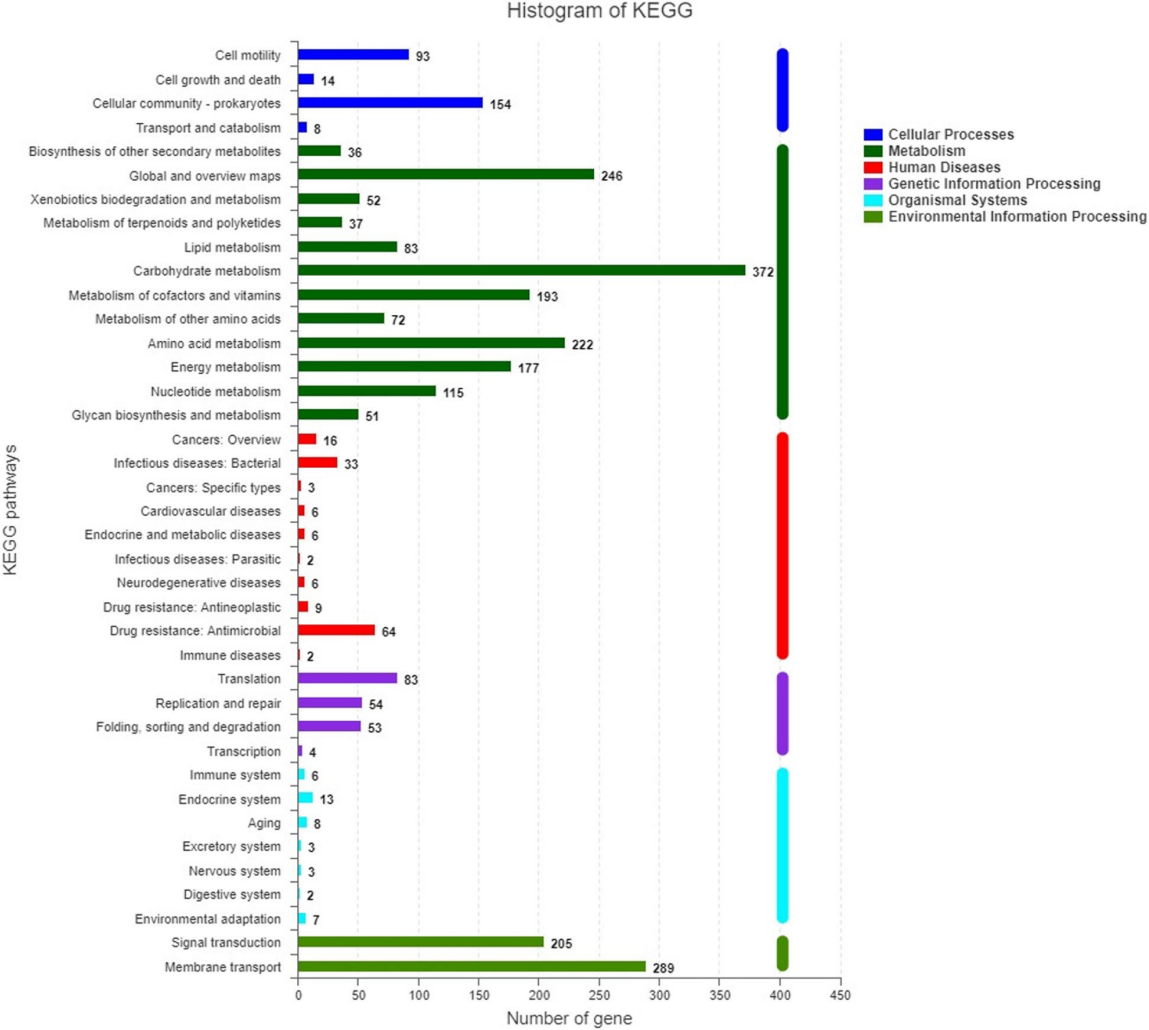
Distribution of functional catalogs of core genes in *Citrobacter* sp. XT1-2-2 after KEGG annotation [30–32]

The analysis of the core and pan genomes showed an uneven distribution among functional categories (Fig. 4). There were several notable differences in the numbers of genes, such as amino acid transport and metabolism (category E), transport and metabolism of carbohydrates (category G), translation (category K) and inorganic ion transport and metabolism (category P). In particular, this difference in the number of genes belonging to the same COG category was mainly reflected in transport and metabolism [5]. For KEGG annotations [30–32], two gene functional categories were enriched in core gene families including metabolism and environmental information processing (Fig. 10). It is noteworthy that the uneven distribution of genes in the COG and KEGG categories was related to transport, metabolism and signal transduction system [18]. The signal transduction systems are responsible for sensing environmental cues and adjusting cellular behavior. Microbial metabolism and transport involve complex metabolic pathway, gene regulation network, and environmental cues. These gene functional categories were enriched among the core gene families in response to complex environmental stimuli. Due to the complex and changeable external environment, strains need to respond quickly to adapt to the environmental changes. So we hypothesized that these gene categories related to transport, metabolism and signal transduction system might provide a competitive advantage to *Citrobacter* sp. XT1-2-2 adapt to the environmental changes.

Determining the taxonomic position is crucial for classifcation, characterization and identifcation of bacteria. The genome of *Citrobacter* sp. XT1-2-2 was submitted to Type Strain Genome Server (TYGS) for whole genome based taxonomic analysis. TYGS compares the query genome with all type strain genomes available in the TYGS database [33] where the intergenomic or intragenomic relations can be inferred through the auto-generated phylogeny and digital DNA-DNA hybridization (dDDH) values. The pairwise comparison between *Citrobacter* sp. XT1-2-2 and the closest type strains using dDDH is shown in Table S12. The table contains dDDH values and confidence intervals for species and subspecies close to *Citrobacter* sp. XT1-2-2 using three different Genome-to-Genome Distance calculator (GGDC) formulas [34].

Whole-genome sequencing technology increased the identification of genomic islands in bacterial genomes. The genomic islands are considered to be the major elements for disseminating resistance genes among bacteria, though the mechanism of transfer was rarely determined [35]. An increasing number of evidences indicated that some genomic islands can transfer between bacteria by conjugation autonomously or with the help of other mobile genetic elements (e.g. conjugative plasmid) [36]. In this study, we discovered a heavy metal resistance genomic island in the chromosome of *Citrobacter* sp. XT1-2-2. The G + C content of *chr*A is 58.87%, and G + C contents of *mer*R, *mer*T, *mer*P, *mer*C, *mer*A, *mer*D and *mer*E are 61.25%,61.25%, 62.68%, 65.37%, 65.68%, 69.70% and 69.20%, respectively, and they differ from that of the XT1-2-2 overall genome (52.09%), suggesting that these resistance genes may be horizontally transferred genes obtained from other bacteria under the stress of external environmental stress.

## Conclusions

Results of comparative genomic analysis from *Citrobacter* sp. XT1-2-2 revealed correlations between genotype and phenotype. Genome analysis revealed all genes of a complete set of sulfate reduction pathway according to the KEGG analysis, which provides the genomic basis for the strain to reduce sulfate (SO_4_^2−^) to sulfide (S^2−^) to form CdS precipitation, thereby reducing the uptake and transport of Cd^2+^ by rice plants. Meanwhile, the strain also revealed various genes responsible for multiple heavy metal resistance, which provided the genomic basis for the strain to adapt to the external complex harmful environment. These analytical results provide insights into the genomic basis of microbial immobilization of heavy metals.

## Materials and methods

### Bacterial strain and DNA extraction

The strain XT1-2-2 was initially isolated from high Cd- contaminated paddy soils (~ 220 mg/kg) in Liuyang city, Hunan Province, China (28°01’N, 113°34’E). Based on previous morphological and molecular characterization, the strain XT1-2-2 was identified as the genus *Citrobacter*. The genomic DNA of the strain XT1-2-2 was extracted by QIAamp DNA Mini Kit (Qiagen, CA, USA) according to the manufacturer’s protocol.

### Bacterial morphological characterization

The selected bacterial strain (*Citrobacter* sp. XT1-2-2) was cultivated in the liquid medium in the absence or presence of 100 mg/L Cd^2+^, and then bacteria were prepared for scanning electron microscopy (SEM), by centrifugation at 12000 rpm for 10 min to pellet bacterial cells. The pellet was resuspended in 4% p-formaldehyde (PFA) to fix the cells for 1 h. Then bacterial cells were resuspended in 200 µl of hexamethyldisilazane (HMDS), and 2 µl suspension was mounted onto a silicon wafer and dried overnight. The samples were investigated using an Quanta 400 FEG (Thermo Scientific, USA) in high-vacuum conditions at 5-kV accelerating voltage.

### Genome sequencing and assembly

Genome sequencing was performed by Shanghai Majorbio Bio-pharm Technology Co., Ltd (Shanghai, China). The genome sequence of the strain XT1-2-2 was obtained via the Illumina Hiseq×10 and Pacbio platforms, with a depth of ~ 100-fold coverage in both platforms. The previously extracted genomic DNA was randomly fragmented through Covaris or Bioruptor method. Fragmented DNA was purified by the QIAquick Nucleotide Removal Kit (Qiagen, Crawley, United Kingdom). Sequencing adaptors were ligated to A-tailed 3’ends according to the manufacturer’s instructions. A library for Illumina Paired-End sequencing was prepared. The sequencing library was sequenced via the combined sequencing method of Illumina Hiseq ×10 + PacBio, and each sample provides at least 100× PacBio sequencing data and 100× Illumina sequencing data of the genome to ensure a more complete and accurate assembly. The constructed standard shotgun library generated 165575 reads totaling 164774594 bp and an average length of 7034.7 bp. The resulting reads were de novo assembled with the help of SOAPdenovo v1.05 [37]. The genome was annotated using the NCBI Pro-karyotic Genome Annotation Pipeline (PGAP), and genes were identified by the gene caller GeneMarkS (Version 4.3). The genomic islands were predicted by Island Viewer 4 [38].

### Identification of gene orthologous groups

OrthoMCL (version 2.0.9) was exploited to determine orthologous families in the pan-genome with default parameter (E-Value: 1e-5, Percent Identity Cutoff: 0, Markov Inflation Index: 1.5). The single-copy core gene and pan gene were extracted with the help of the OrthoMCL (http://www.orthomcl.org/common/downloads/software/v2.0/). Their nucleotide sequences were extracted on the basis of protein ID.

### TYGS analysis

The whole genome sequence of *Citrobacter* sp. XT1-2-2 was uploaded to the Type Strain Genome Server (TYGS) for in silico based taxonomic analysis [33]. The pairwise comparison of the user strain with the type strains were performed using GBDP and accurate intergenomic distances inferred under the “trimming” algorithm and distance formula d5. Digital DDH values and confidence intervals were calculated following the recommended settings of GGDC 2.1 [33]. The intergenomic distances were used to create a balanced minimum evolution tree using FASTME 2.1.4 with 100 pseudobootstrap replicates for branch support [33].

### Core and pan-genome analysis

The comparative study on the core and pan-genome analysis was manipulated by the 16 genome sequences of *Citrobacter* related species according to the previously reported methods [5, 39]. Briefly, the gene set in *Citrobacter sedlakii* NBRC 105722 was selected and regarded as the Reference and the gene sets in the other 15 *Citrobacter* sp. genomes were considered as the Query. The Query genes in each genome were aligned against the Reference genes in reference strain using BLAST v2.2.26 (http://blast.ncbi.nlm.nih.gov/Blast.cgi) and the blast results were filtered by their length and identity. The regression analysis for the core gene cluster curve was performed using a weighted least square regression by fitting the power law n = κexp (m×N) + ϴ to means [40]. N is the number of genomes, n is the number of core gene clusters, ϴ is a constant value representing the predicted minimum number of core genes, and κ and m are parameters.

### Gene functional category

The functional category of the core gene families was analyzed and classified by different database (COG/GO/KEGG). The numbers of corresponding proteins were computed for each term of COG/GO/KEGG.

The main biological functions of different proteins were determined by functional enrichment analysis, and then the resulting results were visualized by GraphPad Prism 7.0.

## Supplementary Information

**Additional file 1:** **Supplementary Table S1**. Classification and general features of *Citrobacter *sp. XT1-2-2.

**Additional file 2:** **Supplementary Table S2.** Project information of *Citrobacter* sp. XT1-2-2.

**Additional file 3:** **Supplementary Table S3.** The genome properties and statistics of *Citrobacter *sp. XT1-2-2

**Additional file 4:** **Supplementary Table S4.** Basic information of sulfate reduction pathwaygenes on Chromosomal and BLASTP analysis in swissprot database.

**Additional file 5:** **Supplementary Table S5.** Basic information of heavy metal resistancegenes on Chromosomal and BLASTP analysis in swissprot database.

**Additional file 6:** **Supplementary Table S6.** all the genes on the genomic islands of *Citrobacter *sp. XT1-2-2.

**Additional file 7:** **Supplementary Table S7.** Basic information of the *Citrobacter* species used for the pan genome analysis.

**Additional file 8:** **Supplementary Table S8.** The core genes in gene category of *Citrobacter *sp. XT1-2-2.

**Additional file 9:** **Supplementary Table S9.** The specific genes in gene category of *Citrobacter *sp. XT1-2-2.

**Additional file 10:** **Supplementary Table S10.** The accessory genes in gene category of *Citrobacter *sp. XT1-2-2.

**Additional file 11:** **Supplementary Table S11.** Classification of the heavy metal resistancegenes in the gene category.

**Additional file 12:** **Supplementary Table S12.** Pairwise digital DNA-DNA hybridization valuesbetween query genome and the selected type strains and whole genomes by Typestrain genome server.

## Data Availability

The genome sequence of *Citrobacter* sp. XT1-2-2 has been deposited in GenBank under the BioSample number SAMN28157541. https://www.ncbi.nlm.nih.gov/biosample/SAMN28157541/.

## Abbreviations

NCBI: National Center for Biotechnology Information
KEGG: Kyoto Encyclopedia of Genes and Genomes
COG: Cluster of Orthologous Groups of Proteins
GBDP: Genome BLAST Distance Phylogeny
dDDH: digital DNA:DNA Hybridization
TYGS: Type Strain Genome Server
PAPS: Phosphoadenylyl-sulphate
HMDS: Hexamethyldisilazane
APS: Adenylylsulphate
GO: Gene Ontology
Cd: Cadmium
