# Supplementary material for: Comparative genomic analysis of Citrobacter sp. XT1-2-2 reveals insights into the molecular mechanism of microbial immobilization of heavy metals
Source: BMC Genomics. 2022 Dec 19;23:838. doi: 10.1186/s12864-022-09069-4 (PMC9764585; doi:10.1186/s12864-022-09069-4)
Supplement: Supplementary file 1 — Additional file 1: Supplementary Table S1. Classification and general features of Citrobacter sp. XT1-2-2. [file 12864_2022_9069_MOESM1_ESM.docx]

Table S1 Classification and general features of *Citrobacter* sp. XT1-2-2

| Property | Term | Evidence code |
| --- | --- | --- |
| Classification | Domain *Bacteria* | TAS[1] |
|  | Phylum *Proteobacteria* | TAS[2] |
|  | Class *Gammaproteobacteria* | TAS[3] |
|  | Order *Enterobacterales* | TAS[4] |
|  | Family *Enterobacteriaceae* | TAS[5] |
|  | Genus *Citrobacter* | TAS[6] |
|  | Species *Citrobacter* sp. | TAS[6] |
| Gram stain | negative | IDA |
| Cell shape | rod | IDA |
| Motility | motile | IDA |
| Sporulation | non-sporulating | IDA |
| Temperature range | 15-40℃ | IDA |
| Optimum temperature | 30℃ | IDA |
| pH range; Optimum | 5-10;6-8 | IDA |
| Habitat | soil | IDA |
| Oxygen requirement | Facultative anaerobic | IDA |
| Biotic relationship | Free-living | IDA |
| Pathogenicity | Non-pathogen | IDA |
| Geographic location | Liuyang city, Hunan province, China | IDA |
| Sample collection | 2016 | IDA |
| Longitude/Latitude | 28°01’N,113°34’E | IDA |

IDA Inferred from Direct Assay, TAS Traceable Author Statement (i.e., a direct report exists in the literature).
