# Supplementary material for: Comparative genomic analysis of Citrobacter sp. XT1-2-2 reveals insights into the molecular mechanism of microbial immobilization of heavy metals
Source: BMC Genomics. 2022 Dec 19;23:838. doi: 10.1186/s12864-022-09069-4 (PMC9764585; doi:10.1186/s12864-022-09069-4)
Supplement: Supplementary file 2 — Additional file 2: Supplementary Table S2. Project information of Citrobacter sp. XT1-2-2. [file 12864_2022_9069_MOESM2_ESM.docx]

Table S2 Project information of *Citrobacter* sp. XT1-2-2

| Property | Term |
| --- | --- |
| Finishing quality | High-quality draft |
| Libraries used | Illumina Paired-End library |
| Sequencing platforms | Illumina Hiseq×10 + Pacbio |
| Fold coverage | 100× |
| Assemblers | SOAPdenovo v1.05 |
| Gene calling method | GeneMarkS+ |
| Project relevance | Bioremediation |
