# Supplementary material for: Comparative genomic analysis of Citrobacter sp. XT1-2-2 reveals insights into the molecular mechanism of microbial immobilization of heavy metals
Source: BMC Genomics. 2022 Dec 19;23:838. doi: 10.1186/s12864-022-09069-4 (PMC9764585; doi:10.1186/s12864-022-09069-4)
Supplement: Supplementary file 3 — Additional file 3: Supplementary Table S3. The genome properties and statistics of Citrobacter sp. XT1-2-2 [file 12864_2022_9069_MOESM3_ESM.docx]

Table S3 The genome properties and statistics of *Citrobacter* sp. XT1-2-2

| Attribute | Value |
| --- | --- |
| Genome size (bp) | 5040459 |
| CDS No. | 4681 |
| G+C Content (%) | 52.09 |
| tRNA No. | 84 |
| ncRNAs No. | 11 |
| rRNA No. | 25 |
| Gene No. | 4801 |
| Pseudo Genes (total) | 80 |
| CDSs (with protein) | 4601 |
| GC content in gene region (%) | 53.11 |
| Intergenetic region length (bp) | 580512 |
| GC content in intergenetic region (%) | 44.19 |
| Intergenetic length/Genome (%) | 11.52 |
| Total reads num | 165575 |
| Average length of reads(bp) | 7034.7 |
| Bases in all scaffolds(bp) | 5040459 |
| Scaffold N50(bp) | 5040459 |
| Scaffold N90(bp) | 5040459 |
| CRISPR-Cas No. | 2 |
| Total lengthof tandem repeat (bp) | 6530 |
| Tandem repeat /Genome (%) | 0.13 |
| Genes No. of Cellular Component | 2053 |
| Genes No. of Molecular Function | 3098 |
| Genes No. of Biological Process | 3098 |
| Genes assigned to COGs | 4383 |
| Genes with Pfam domains | 4294 |
| Genes with transmembrane helices | 1170 |
| Genes with transport proteins | 1195 |
| Genes with signal peptides | 416 |
