# Supplementary material for: Comparative genomic analysis of Citrobacter sp. XT1-2-2 reveals insights into the molecular mechanism of microbial immobilization of heavy metals
Source: BMC Genomics. 2022 Dec 19;23:838. doi: 10.1186/s12864-022-09069-4 (PMC9764585; doi:10.1186/s12864-022-09069-4)
Supplement: Supplementary file 4 — Additional file 4: Supplementary Table S4. Basic information of sulfate reduction pathwaygenes on Chromosomal and BLASTP analysis in swissprot database. [file 12864_2022_9069_MOESM4_ESM.docx]

Table S4 Basic information of sulfate reduction pathway genes on Chromosomal and BLASTP analysis in swissprot database

| Gene ID on chromosome | Gene Name | Gene Description | Protein ID in database | Protein Description |
| --- | --- | --- | --- | --- |
| Gene0991 | *cys*J | Sulfite reductase subunit alpha | Q57KH7.3 | Sulfite reductase [NADPH] flavoprotein alpha |
| Gene0992 | *cys*I | Sulfite reductase subunit beta | A9N2E5.1 | Sulfite reductase [NADPH] hemoprotein beta |
| Gene0993 | *cys*H | Phosphoadenosine phosphosulfate reductase | A8ANW8.1 | Phosphoadenosine 5'-phosphosulfate reductase |
| Gene0994 | *iap* | Zn-dependent exopeptidase M28 | P10423.1 | Alkaline phosphatase isozyme conversion protein |
| Gene0995 | *cys*D | Sulfate adenylyltransferase subunit 2 | Q0TEA6.1 | Sulfate adenylyltransferase subunit 2 |
| Gene0996 | *cys*N | Sulfate adenylyltransferase | A8ANW5.1 | Sulfate adenylyltransferase subunit 1 |
| Gene0997 | *cys*C | Adenylyl-sulfate kinase | A1AEU4.1 | Adenylyl-sulfate kinase |
| Gene1439 | *cys*P | Thiosulfate transporter subunit | P16700.1 | Thiosulfate-binding protein |
| Gene1440 | *cys*U | Sulfate/thiosulfate transporter subunit | P16701.1 | Sulfate transport system permease protein CysT |
| Gene1441 | *cys*W | Sulfate/thiosulfate transporter permease subunit | P0AEB0.1 | Sulfate transport system permease protein CysW |
| Gene1442 | *cys*A | Sulfate/thiosulfate transporter subunit | P16676.2 | Sulfate/thiosulfate import ATP-binding protein CysA |
