# Supplementary material for: Comparative genomic analysis of Citrobacter sp. XT1-2-2 reveals insights into the molecular mechanism of microbial immobilization of heavy metals
Source: BMC Genomics. 2022 Dec 19;23:838. doi: 10.1186/s12864-022-09069-4 (PMC9764585; doi:10.1186/s12864-022-09069-4)
Supplement: Supplementary file 5 — Additional file 5: Supplementary Table S5. Basic information of heavy metal resistancegenes on Chromosomal and BLASTP analysis in swissprot database. [file 12864_2022_9069_MOESM5_ESM.docx]

Table S4 Basic information of heavy metal resistance genes on Chromosomal and BLASTP analysis in swissprot database

| Gene ID on chromosome | Gene Name | Gene Description | Protein ID in database | Protein Description |
| --- | --- | --- | --- | --- |
| gene3258 | *czc*D | zinc transporter ZitB | Q8ZQT3.1 | Zinc transporter ZitB |
| gene3438 | *chr*A | chromate transporter | Q58128.1 | Uncharacterized transporter MJ0718 |
| Gene0278 | *znt*A | zinc/cadmium/mercury/lead-transporting ATPase | Q3YW59.1 | Zinc/cadmium/lead-transporting P-type ATPase |
| Gene2042 | *znu*B | zinc ABC transporter permease | P39832.3 | High-affinity zinc uptake system membrane protein ZnuB |
| Gene2043 | *znu*C | zinc ABC transporter ATP-binding protein ZnuC | Q57NA5.2 | Zinc import ATP-binding protein ZnuC |
| Gene2044 | *znu*A | zinc ABC transporter substrate-binding protein | P39172.4 | High-affinity zinc uptake system protein ZnuA |
| Gene1379 | *ars*C | arsenate reductase | P76569.1 | Uncharacterized protein YfgD |
| Gene4560 | *ars*B | arsenical pump membrane protein | P74985.1 | Arsenical pump membrane protein |
| Gene4561 | *ars*H | arsenical resistance protein ArsH | Q92R45.1 | NADPH-dependent FMN reductase ArsH |
| *Gene3508* | *cusA* | cation transporter | Q8FK36.1 | Cation efflux system protein CusA |
| Gene3509 | *cus*B | copper resistance protein | P77239.1 | Cation efflux system protein CusB |
| Gene3510 | *cus*F | copper-binding protein | Q8CWA3.1 | Cation efflux system protein CusF |
| Gene3511 | *cus*C | copper transporter | Q8XBY3.1 | Cation efflux system protein CusC |
| *Gene3513* | *cusS* | two-component sensor histidine kinase | P77485.1 | Sensor histidine kinase CusS |
| Gene3421 | *mer*R | Hg(II)-responsive transcriptional regulator | P0A2Q8.1 | Mercuric resistance operon regulatory protein |
| Gene1982 | *mer*T | membrane protein | Q57347.1 | Uncharacterized protein HI_1049 |
| Gene1983 | *mer*P | heavy metal transporter | / | No significant similarity found |
| Gene3424 | *mer*C | mercury transporter MerC | Q50919.1 | Mercuric transport protein MerC |
| Gene3425 | *mer*A | mercuric reductase | P08332.1 | Mercuric reductase |
| Gene3426 | *mer*D | mercuric reductase (plasmid) | P20102.1 | HTH-type transcriptional regulator MerD |
| Gene3427 | *mer*E | mercury resistance protein | Q7AKA4.1 | Broad mercury transporter MerE |
| Gene1901 | *cbi*G | cobalamin biosynthesis protein CbiG | Q05631.1 | Cobalt-precorrin-5A hydrolase |
| Gene1902 | *cob*J | cobalt-precorrin-3B C(17)-methyltransferase | Q05590.1 | Probable cobalt-factor III C(17)-methyltransferase |
| Gene1903 | *cob*K-*cbi*J | cobalt-precorrin-6A/precorrin-  6x reductase | Q05591.1 | Cobalt-precorrin-6A reductase |
| Gene1904 | *cbi*K | sirohydrochlorin cobaltochelatase | Q05592.1 | Sirohydrochlorin cobaltochelatase |
| Gene1905 | *cob*I-*cbi*L | precorrin-2 C(20)-methyltransferase | Q05593.2 | Cobalt-precorrin-2 C(20)-methyltransferase |
| Gene1906 | *cbi*M | cobalamin biosynthesis protein CbiM | Q05594.1 | Cobalt transport protein CbiM |
| Gene1907 | *cbi*N | cobalt ABC transporter substrate-binding protein CbiN | A9MLS2.1 | Cobalt transport protein CbiN |
| Gene1908 | *cbi*Q | cobalt transporter CbiQ | Q05598.2 | Cobalt transport protein CbiQ |
| Gene1909 | *cbi*O | cobalt transporter ATP-binding subunit | Q5PDU4.1 | Cobalt import ATP-binding protein CbiO |
| Gene1910 | *cob*Q | cobyric acid synthase CobQ | A8AEQ8.1 | Cobyric acid synthase |
| Gene1911 | *cob*P | adenosylcobinamide kinase/adenosylcobinamide phosphate guanyltransferase | Q05599.2 | Bifunctional adenosylcobalamin biosynthesis protein CobU |
| Gene1912 | *cob*S | adenosylcobinamide-GDP ribazoletransferase | B4T8W9.1 | Adenosylcobinamide-GDP ribazoletransferase |
| Gene1913 | *cob*U | nicotinate-nucleotide--dimethylbenzimidazole phosphoribosyltransferase | B4TZP5.1 | Nicotinate-nucleotide--dimethylbenzimidazole phosphoribosyltransferase |
