# Supplementary material for: Comparative genomic analysis of Citrobacter sp. XT1-2-2 reveals insights into the molecular mechanism of microbial immobilization of heavy metals
Source: BMC Genomics. 2022 Dec 19;23:838. doi: 10.1186/s12864-022-09069-4 (PMC9764585; doi:10.1186/s12864-022-09069-4)
Supplement: Supplementary file 7 — Additional file 7: Supplementary Table S7. Basic information of the Citrobacter species used for the pan genome analysis. [file 12864_2022_9069_MOESM7_ESM.docx]

Table S6 Basic information of the *Citrobacter* species used for the pan genome analysis

| Strain name | Accession number | GC% | Protein count | Genome size (bp) |
| --- | --- | --- | --- | --- |
| *Citrobacter portucalensis* P10159 | CP012554.1 | 51.7 | 4867 | 5080321 |
| *Citrobacter freundii* B38 | CP016762.1 | 51.7 | 4938 | 5134500 |
| *Citrobacter freundii* SL151 | CP016952.1 | 51.7 | 4752 | 5096586 |
| *Citrobacter freundii* ST1 | KQ464178.1 | 51.7 | 3192 | 3448390 |
| *Citrobacter koseri* ATCC BAA-895 | NC_009792.1 | 53.9 | 4269 | 4720462 |
| *Citrobacter rodentium* ICC168 | NC_013716.1 | 54.8 | 4946 | 5346659 |
| *Klebsiella aerogenes* KCTC 2190 | NC_015663 | 54.8 | 4876 | 5280350 |
| *Citrobacter youngae* ATCC 29220 | NZ_ABWL00000000.2 | 52.69 | 4741 | 5150259 |
| *Citrobacter sedlakii* NBRC 105722 | NZ_BBNB00000000.1 | 54.72 | 4266 | 4631466 |
| *Citrobacter pasteurii* CIP 55.13 | NZ_CDHL00000000.1 | 51.8 | 4324 | 4986623 |
| *Citrobacter amalonaticus* Y19 | NZ_CP011132.1 | 53.4 | 5182 | 5584358 |
| *Citrobacter werkmanii* BF-6 | NZ_CP019986.1 | 52.1 | 4587 | 4929789 |
| *Citrobacter braakii*  FDAARGOS_253 | NZ_CP020448.2 | 52.0 | 4757 | 5244290 |
| *Citrobacter farmeri* AUSMDU00008141 | NZ_CP022695.1 | 53.4 | 4814 | 5130228 |
| *Citrobacter freundii* 97/79 | NZ_FLYB00000000.1 | 51.89 | 4875 | 5293620 |
| *Citrobacter* sp. XT1-2-2 | CP097324.1 | 52.09 | 4664 | 5040459 |
