# Supplementary material for: Comparative genomic analysis of Citrobacter sp. XT1-2-2 reveals insights into the molecular mechanism of microbial immobilization of heavy metals
Source: BMC Genomics. 2022 Dec 19;23:838. doi: 10.1186/s12864-022-09069-4 (PMC9764585; doi:10.1186/s12864-022-09069-4)
Supplement: Supplementary file 11 — Additional file 11: Supplementary Table S11. Classification of the heavy metal resistancegenes in the gene category. [file 12864_2022_9069_MOESM11_ESM.docx]

Table S10 Classification of the heavy metal resistance genes in the gene category

| Gene Category | Names of heavy metal resistance genes on the chromosome of strain XT1-2-2 |
| --- | --- |
| Core Genes | *czc*D, *znt*A, *znu*B, *znu*C, *znu*A, *ars*C, *cob*P, *cob*S, *cob*U. |
| Accessory Genes | *chr*A, *ars*B, *ars*H, *cus*A, *cus*B, *cus*F, *cus*C, *cus*S, *mer*R, *mer*T, *mer*P, *mer*C, *mer*A, *mer*D, *mer*E, *cbi*G, *cob*J, *cob*K-*cbi*J, *cbi*K, *cob*I-*cbi*L, *cbi*M, *cbi*N, *cbi*Q, *cbi*O, *cob*Q. |
| Unique Genes | Not found |
